# Contributing Factors to Sensorimotor Adaptability in Reactive Agility Performance in Youth Athletes

**DOI:** 10.2478/hukin-2022-0067

**Published:** 2022-09-08

**Authors:** Teresa Zwierko, Anna Nowakowska, Wojciech Jedziniak, Marek Popowczak, Jarosław Domaradzki, Joanna Kubaszewska, Mariusz Kaczmarczyk, Andrzej Ciechanowicz

**Affiliations:** 1 Institute of Physical Culture Sciences, Functional and Structural Human Research Centre, University of Szczecin, Szczecin, Poland.; 2 Department of Team Games, Faculty of Physical Education and Sport, University School of Physical Education in Wroclaw, Wroclaw, Poland.; 3 Department of Biostructure, Faculty of Physical Education and Sport, University School of Physical Education in Wroclaw, Wroclaw, Poland.; 4 Department of Clinical and Molecular Biochemistry, Pomeranian Medical University, Szczecin, Poland.

**Keywords:** gender, maturity, age, brain-derived neurotrophic factor, team sport games

## Abstract

Sensorimotor adaptability facilitates adjusting behaviour for changing environmental stimuli to maintain appropriate goal-directed motor performance. Its effectiveness is associated with perceptual-cognitive modulation. As the factors affecting it are still not completely known, the aim of our study was therefore to analyse the association between selected variables (demographic, training, anthropometric, genetic) and sensorimotor adaptation in reactive agility tasks in youth team-sport athletes. The study group consisted of 85 youth athletes (aged 12.61 ± 0.98 years). Based on an initial evaluation, participants were divided into faster and slower agility groups. The resultant differences between change of direction speed tests and reactive agility tests provided the REAC-INDEX as a dependent variable. The independent variables were as follows: gender, calendar age, body mass, height, BMI, maturity offset, training status and the BDNF rs6265 polymorphism. Multiple linear regression showed that the maturity offset (ß = 0.269; p = 0.012) and calendar age (ß = -0.411; p < 0.001) significantly contributed to the REAC-INDEX of all participants (R^2^ = 0.13). In the slower group, the c.196G BDNF allele had a significant influence (ß = -0.140; p = 0.044) on the REAC-INDEX. The best predictive model comprised female gender (ß = 0.799; p < 0.001), maturity offset (ß = -0.586; p < 0.001) and training experience (ß = -0.225; p = 0.009), contributing to 49% of RA variance. Sensorimotor adaptability is mainly dependent on gender and age, and can be improved through systematic sports training. The BDNF rs6265 polymorphism may be considered a contributing factor to SA variability in the initial stages of training, although polymorphism-related differences blurred as the effect of participation in sports training increased.

## Introduction

Agility in sport is broadly defined as a rapid whole-body movement with changes in velocity or direction in response to stimuli (Sheppard and Young, 2006). Based on this definition, two aspects of agility are listed, i.e., the rate of change of direction (COD) and reactive agility (RA). COD while sprinting is usually based on patterns, and so is classified as a pre-planned and closed skill, and is usually defined as the ability to change direction in the shortest time possible within a predetermined location and space (Young et al., 2015), determined generally by physical attributes (Freitas et al., 2019).

In contrast, RA refers to the non-planned ability to perform a change in direction or speed in response to external stimuli (Spiteri et al., 2018). This implies that cognitive and perceptual factors are significant facets of RA (Paul et al., 2016). The relationship between perception and movement in RA tasks indicates the necessity for an athlete to formulate adaptive movement responses to environmental constraints (Spiteri et al., 2018). Although perceptual-cognitive functions have been identified as a dependent factor in achieving faster agility performance, the factors behind the adaptation mechanisms of athletes to appropriate movement direction and producing a faster performance are currently unknown.

Sensorimotor adaptation (SA) based on cognitive and perceptual factors seems to be a major discriminating factor between performance in RA and COD. SA involves adjusting motor commands in tasks, resulting in an updated forward modelling process of the motor control system (Seidler and Carson, 2017), making it possible to adjust behaviour to changing environmental or internal demands to maintain appropriate goal-directed motor performance (Ruitenberg et al., 2018). It has been reported that the effectiveness of sensorimotor adaptation is associated with cortical modulation and plasticity. Neural plasticity is specifically required to maintain proper sensorimotor integration in the interface between the sensory and motor systems in reactivity motor tasks, when players react in a dynamically changing, unpredictable and fast-paced environment. This phenomenon could be explained by the adaptation functions related to high sensorimotor activity during systematic participation in reactive situations in sports (Hülsdünker et al., 2018).

The experimental study results suggest that the brain-derived neurotrophic factor (BDNF) may be a key modulator of cortical plasticity in motor circuits (McHughen et al., 2011). BDNF, the most abundant and widely distributed neurotrophin in the mammalian central nervous system, is encoded by a gene located on chromosome 11p14.1. The *BDNF* rs6265 (c.196G>A) polymorphism, which results in a change of valine (Val) to methionine (Met) at amino acid position 66 (p.Val66Met) of the BDNF protein, was associated with impaired activity-dependent secretion of this neurotrophin (Egan et al., 2003). The results of previous studies have suggested that the *BDNF* rs6265 polymorphism has an inhibitory effect on sensorimotor adaptability in fine motor skills; however, those experimental findings were not conclusive (Barton et al., 2014; Gonzalez-Giraldo et al., 2016; Joundi et al., 2012).

While expert-novice differences in agility tasks have been well documented (Paul et al., 2016; Spiteri et al., 2018) as mainly the effect of an improved ability to identify task relevant cues and differentiate between various sources of information to produce an accurate and rapid motor response, little is known about this functioning across age and gender, anthropometric variables, sports experience and genetic conditioning. In the current study, we attempted to explore the association between SA in reactive agility and selected variables (demographic, training, anthropometric and genetic (*BDNF* rs6265 polymorphism)) in youth team-sport athletes. In this study we refer to SA as the difference between RA and COD (representing the time required for reactive processes to occur), and defined it as REAC-INDEX (Fiorilli et al., 2017). We hypothesized that SA in reactive agility tasks would vary by gender (Armstrong et al., 2001) and improve with age and sports experience (Fiorilli et al., 2017; Lloyd et al., 2015), as a physical development required to master perceptual-cognitive skills and motor demands in a specific sport domain. Alternatively, in line with findings showing an association of BDNF genetic variants with cortical plasticity in a sensorimotor task (Gonzalez-Giraldo et al., 2016; Joundi et al., 2012; Kleim et al., 2006; McHughen et al., 2011), we hypothesized that sensorimotor adaptation may also be associated with the *BDNF* 196 G/A polymorphism.

## Methods

### Participants

The study group consisted of 85 youth athletes (42 girls and 43 boys, aged 12.61 ± 0.98 years) systematically practising team sports, i.e., volleyball (n = 25), soccer (n = 41) and basketball (n = 19). The mean sport experience was 3.35 ± 0.93 years. Based on an initial evaluation of SA, participants were divided into faster and slower agility groups. Participants up to the 50^th^ percentile (median time) were assigned to the faster group, and participants beyond the 50^th^ percentile were assigned to the slower group. The study was approved by the Local Bioethics Committee in Szczecin (No. 03/KB/VII/2019) and conducted in accordance with the ethical principles for medical research involving human subjects by the World Medical Association, as outlined in the Declaration of Helsinki. All participants and their parents provided a written informed consent form prior to the study.

### Anthropometry

Standing height and sitting height measurements were made with accuracy of 0.1 cm using an anthropometer (GPM Anthropological Instruments). Body mass was measured in kilograms using an electronic scale (Seca 769, Seca, Hamburg, Germany). These data were then incorporated into a regression equation to predict a maturity offset, comprising the length of time (in years) from peak height velocity (PHV) (Mirwald et al., 2002): boys’ maturity offset = - 9.236 + 0.0002708 x (leg length and sitting height interaction) + 0.001663 x (age and leg length interaction) + 0.007216 x (age and sitting height interaction) + 0.02292 x (weight by height ratio); girls’ maturity offset = -9.276 + 0.0001882 x (leg length and sitting height interaction) + 0.0022 x (age and leg length interaction) + 0.005841 x (age and sitting height interaction) + 0.02658 x (age and weight interaction) + 0.07693 x (weight by height ratio).

### Agility and Sensorimotor Adaptability Assessment

Before the start of agility testing, participants underwent a standardised 15 min warm-up procedure. A Fusion Smart Speed System (Fusion Sport, Coopers Plains, QLD, Australia) was used during the ‘five-time shuttle run to gates’ test for the determination of the change of direction speed and reactive agility. The system was comprised of electronic gates with a photocell with an infrared transmitter and a light reflector, a Smart Jump mat integrated with a photocell, an RFID reader for identification of the athletes' tag, and computer software. The testing apparatus measured the running time with accuracy of 0.001 s. Data from the tests were recorded on a PDA (HP iPAQ 112) with the participant's name. The “five-time shuttle run to gates” test allowed measurement of the time of movement during repeated “stop’n’go” directional changes in response to a light signal. The Fusion Smart Speed System application was used for fixed (pre-planned) and random selections of the lamp signal at the gate, following the procedures proposed by Popowczak et al. (2016). All participants ran five times from the starting mat to the particular gates (lines placed between photocells with reflectors, 1 m long) and then to the next gate's mat. The mats at the starting and finishing lines had integral photocells. As soon as both of the participant’s feet were in contact with the central part of the mat, the participant received a light signal indicating the next gate they should run to. In the rate-of-change-of-direction test (COD), athletes ran to the gates in a fixed sequence. The COD test was repeated twice with a 3-min rest interval in between and the best result was used for further analysis. In the reactive agility test, a run to randomly selected gates was recorded. The order was different for each run, and all athletes covered the same distance. The RA test was repeated twice with a 3-min rest interval in between, and the best result (total time) of the run was used for further analysis. For the SA evaluation, a REAC-INDEX was calculated (Fiorilli et al., 2017), representing the time difference between the result of the RA test and the COD test, according to the formula: REAC-INDEX [s] = RA [s] – COD [s]. Based on the individual REAC-INDEX, participants were divided into two groups of fast and slow reactions. To this end, a median REAC-INDEX was calculated. If a participant's specific REAC-INDEX was below or equal to the overall median, then that time was labelled in the dataset as fast, and if it was beyond the median time, then it was categorized as slow.

### BDNF Genotyping

Genomic DNA was extracted from buccal epithelial cells using a commercially available DNA isolation kit (QIAamp DNA Mini Kit; QIAGEN, Hilden, Germany) according to the manufacturer’s protocol. All samples were independently genotyped using a blind method, in duplicate, i.e., all samples were anonymously labelled by one person and then genotyped by a second person. The SNP was genotyped using an allelic discrimination assay on a CFX96 Touch Real-Time PCR Detection System (Bio-Rad, Germany) with TaqMan® probes. To discriminate BDNF rs6265 alleles, TaqMan® Pre-Designed SNP Genotyping Assays were used (Thermo Fisher Scientific Inc., USA). The assay ID of SNP was C_11592758_10, including primers and fluorescently labelled (FAM and VIC) MGB™ probes to detect alleles.

### Statistical Analysis

The distribution of each quantitative variable was tested for skewness with the Shapiro-Wilk test. Quantitative data were presented as medians. The basic characteristics and agility performance of participants in regard to gender were compared using the Mann-Whitney test. Possible divergence of BDNF genotype frequencies from the Hardy-Weinberg equilibrium was assessed using a χ^2^ test with 1 degree of freedom. Differences in the frequencies of genotypes and alleles between girls and boys were tested for statistical significance using a χ^2^ test or a Fisher’s exact test if necessary. A backward multiple linear regression analysis with an adjustment for gender was performed to identify the relationships between sensorimotor adaptability (dependent variable) and the remaining independent variables (continuous and categorical). Statistical significance was defined at the level of *p* < 0.05.

## Results

Gender-specific analyses revealed that boys had lower morphological variables compared to girls, specifically in regard to body mass (*p* = 0.004), body height (*p* = 0.006) and BMI (*p* = 0.016) (Table 1). A significant difference in maturity offset between the groups was also found. On average, boys presented a negative offset, which means that they had not yet reached PHV, i.e., they were about 1.1 years prior to experiencing peak height velocity, whereas the group of girls presented a positive (0.8 < 1.5) offset, indicating that they were within their PHV period.

**Table 1:** Basic characteristics and quantitative measures of agility by gender

Variable	Females (n = 42)	Males (n = 43)	*p*
Calendar age [years]	12.4 (11.2-13.6)	12.5 (11.5-13.4)	0.258
Body mass [kg]	52.9 (37.7-72.6)	45.4 (31.5-69.0)	0.004
Body height [cm]	165.05 (145.55-183.65)	157.2 (142.3-180.5)	0.006
BMI	20.14 (15.45-25.47)	17.97 (15.05-23.72)	0.016
Maturity Offset [+/-]	0.8 (-1.9-4.5)	-1.1 (-2.5-0.8)	< 0.000
GG *BDNF* [%]	73.8	79.1	0.404^#^
GA+AA *BDNF* [%]	26.2	20.9	
Training experience [years]	3.0 (2.0-4.0)	4.0 (2.0-5.0)	0.007
CODs [s]	17.25 (14.88-21.66)	16.24 (14.63-21.48)	< 0.000
RA[s]	20.05 (17.69-24.11)	18.51 (16.96-24.67)	< 0.000
REAC-INDEX [s]	2.70 (1.34-4.34)	2.29 (-2.75-4.40)	0.002

Note. Data are presented as medians (minimum-maximum), ^#^ p for χ^2^ test, CODs - change-of-direction speed test, RA - reactive agility test, REAC-INDEX - difference between the RA and CODs tests

There were 65 GG *BDNF* homozygotes (76.5%), 17 GA heterozygotes (20.0%) and 3 AA homozygotes (3.5%) in the studied group. The frequency of the minor c.196A *BDNF* allele was 13.5%. The c.196G>A *BDNF* genotype distribution conformed to the expected Hardy-Weinberg equilibrium (*p* = 0.181). In the female subgroup, there were 31 GG *BDNF* homozygotes (73.8%) and 11 GA heterozygotes (26.2%), with 13.1% frequency of the minor c.196A *BDNF* allele. In the male subgroup, there were 34 GG *BDNF* homozygotes (79.1%), 6 GA heterozygotes (13.9%) and 3 AA homozygotes (7.0%), with 13.9% frequency of the *BDNF* major G allele. No significant differences in the distribution of the rs6265 *BDNF* genotypes or alleles were found between both genders.

Compared to girls, boys obtained better results in all agility tests, i.e., in the rate-of-change-of-direction test (COD, *p* < 0.001), and in the reactive agility test, as well as a shorter time for sensorimotor adaptation regarding the REAC-INDEX.

For the analysis of the contributing factors to sensorimotor adaptability in reactive agility performance in youth athletes, a multiple linear regression analysis for all participants was used. Next, based on the individual REAC-INDEX, results were analysed according to the group, faster or slower. To identify the contribution of independent variables in the sensorimotor adaptability, we created models for REAC-INDEX and RA (dependent variables).

A stepwise backward multiple regression analysis was used to eliminate independent variables from the initial models and obtain the best predictive models for REAC-INDEX, each contributing to 13% of the variance of REAC-INDEX for all youth athletes and 25% of the variance of REAC-INDEX for the slower group. REAC-INDEX score distribution for the faster group was bimodal in nature; therefore, in this case a regression model was not created. The best predictive models for RA contributed to 49% of the variance in RA for all the youth athletes, 54% of the variance in RA for the fast group and 45% of the variance in RA for the slow group (Table 2).

**Table 2:** Adjusted coefficients of determination in the best predictive models for REAC-INDEX and RA

Model	R	R^2^	Adjusted R^2^	Residual statistics	
				F	*p*
**All participants**					
REAC-INDEX ^a^	0.386	0.149	0.128	7.185	0.001
RA ^aa^	0.714	0.509	0.491	28.021	< 0.000
**Faster group**					
REAC-INDEX ^b^	unavailable				
RA ^bb^	0.768	0.590	0.546	13.652	< 0.000
**Slower group**					
REAC-INDEX ^c^	0.537	0.288	0.251	7.906	0.001
RA ^cc^	0.693	0.480	0.454	18.017	< 0.000

Note. Dependent variables: REAC-INDEX, RA ^a^ Predictors for REAC-INDEX of all participants: calendar age, maturity offset; ^aa^ predictors for RA of all participants: gender, maturity offset, training experience ^b^ Predictors for REAC-INDEX of the fast group: unavailable; ^bb^ predictors for RA of the fast group: maturity offset, training experience, BMI, gender ^c^ Predictors for REAC-INDEX of the slow group: calendar age, c.196G BDNF polymorphism; ^cc^ Predictors for RA of the slow group: maturity offset, gender

The standardized regression coefficient values (ß) for the independent variables in the best predictive models for REAC-INDEX are shown in Table 3. Maturity offset (ß = -0.269; *p* = 0.012) and calendar age (ß = -0.411; *p* < 0.000) significantly contributed to the REAC-INDEX of each participant. Calendar age (ß = -0.378; *p* = 0.009) and c.196G *BDNF* genotype (ß = -0.140; *p* = 0.044) had a significant influence on the REAC-INDEX of the slower group, which means that in this case, a shorter REAC-INDEX was achieved by older athletes and those with wild-type homozygotes (GG).

**Table 3:** The B and ß coefficients of the best predictors for REAC-INDEX

Model	B	ß	95% CI		t	*p*
**REAC-INDEX of all participants**						
Constant	8.334	-	-	-	5.269	< 0.000
Maturity offset	-0.111	-0.269	-0.066	-0.516	-2.569	0.012
Calendar age	-0.038	-0.411	-0.636	-0.186	-3.629	<0.000
**REAC-INDEX of the slow group**						
Constant	6.760	-	-	-	5.217	< 0.000
Calendar age	-0.024	-0.378	-0.662	-0.094	2.689	0.009
c.196G *BDNF*	-0.177	-0.140	-0.576	-0.008	2.079	0.044

The standardized regression coefficient values (ß) for the independent variables in the best predictive models for RA are shown in Table 4. Female gender (ß = 0.799; *p* < 0.000), a negative maturity offset in regard to the PHV period (ß = - 0.586; *p* < 0.000), and shorter training experience (ß = -0.225; *p* = 0.009) negatively affected RA. The model for RA of the faster group, next to the variables of female gender (ß = 0.844; *p* < 0.000), negative maturity offset (ß = -0.598; *p* < 0.000) and shorter training experience (ß = -0.275; *p* = 0.020), also included BMI (ß = 0.265; *p* = 0.045). In turn, the model for RA of the slower group consisted of only two independent variables, i.e., female gender (ß = 0.824; *p* < 0.000) and negative maturity offset (ß = -0.806; *p* < 0.000).

**Table 4:** The B and ß coefficients of the best predictors for RA

Model	B	ß	95% CI		t	*p*
**RA of all participants**						
Constant	20.910	-	-	-	41.935	< 0.000
Gender (female)	1.323	0.799	0.579	1.019	7.229	< 0.000
Maturity offset	-0.562	-0.586	-0.794	-0.378	-5.609	< 0.000
Training experience	-0.384	-0.225	-0.392	-0.058	-2.676	0.009
**RA of the faster group**						
Constant	16.928	-	-	-	9.481	< 0.000
Gender (female)	1.214	0.844	0.557	1.131	5.948	< 0.000
Maturity offset	-0.497	-0.598	-0.913	-0.284	-3.855	< 0.000
Training experience	-0.425	-0.275	-0.505	-0.045	- 2.421	0.020
BMI	0.201	0.265	0.006	0.525	2.068	0.045
**RA of the slower group**						
Constant	20.027	-	-	-	97.678	< 0.000
Gender (female)	1.460	0.824	0.520	1.127	5.489	< 0.000
Maturity offset	-0.796	-0.806	-1.110	-0.503	5.375	< 0.000

## Discussion

In the present study, we analysed the effect of selected demographic, training, anthropometric and genetic (*BDNF* rs6265 polymorphism) variables on SA in a reactive agility (RA) test in youth team-sport athletes. Our hypothesis that SA in an agility task varied by gender and improved with athletes’ age and sport experience, as a physical development required for mastering perceptual-cognitive skills and motor demands in the specific sport domain, was confirmed. Gender, maturity offset and training experience contributed to 49% of the variance in RA for the entire study, with the variance even higher in the faster group (54%).

It appears that gender difference, in terms of all aspects of agility performances, is manifested in individual child development, and is associated with different rates of biological maturation. A previous study showed no significant gender differences in the agility testing of 6 to 9 year-old children (Yanci et al., 2014). In contrast, in children aged 9 to 12 years, it has been observed that boys outperformed girls in several physical fitness tests, including the agility star run test (moderate effect size, d = 0.48) (Golle et al., 2015). Moreover, significantly better agility of boys was confirmed in a 4 x 5 m shuttle run test in Polish children aged 4–7 years (Przednowek et al., 2021). The better agility performance of boys can be explained by their higher absolute and relative anaerobic power. Peak power and mean power in the Wingate anaerobic test for boys were higher than for the girls, and this gender difference increased with age, i.e., peak anaerobic power of boys increased by 121% and mean power by 113% from age 12 to 17 years, while those of girls increased by 66% and 60%, respectively (Armstrong et al., 2001). The study also showed curvilinear improvements in agility with age, namely accelerated improvements in agility for boys aged 9–10 years and for girls aged 9–11 years (Golle et al., 2015). Furthermore, in our study, boys were on average 1.1 years prior to experiencing peak height velocity (PHV), while girls presented a positive (0.8) offset, indicating that they were within the PHV period. The effects of PHV consist of anatomical and functional changes as well as changes of proportions in tissue composition of the body. In consequence, a different growth rate should affect gender differentiation in somatic features, the somatotype and motor abilities.

The present study showed that maturity offset and calendar age were also the most significant factors contributing to SA of all participants. Sensorimotor adaptation involves visuospatial cognitive processes, specifically for the processing of speed and response inhibition, that reach maturity by 14-15 years of age (Luna et al., 2004), simultaneously to the development of agility (Lloyd et al., 2015). It seems that sensorimotor adaptability in reactive tasks can be improved by participation in fast-paced sports. Moreover, experimental studies show that perceptual-cognitive processes associated with agility performance are indeed trainable (Hülsdünker et al., 2018; Spiteri et al., 2018).

An analysis of the predictive model of the REAC-INDEX in the slower agility group revealed that a 25% decrease in the REAC-INDEX (higher values suggest slower processing speed) was associated with a higher age and the c.196G *BDNF* polymorphism (wild-type homozygotes). In light of the above, our second hypothesis that sensorimotor adaptation may be also associated with the c.196G>A *BDNF* polymorphism was partly confirmed.

Physical exercise has been shown to enhance brain functioning in children, in turn leading to better neurocognitive processing (Ludyga et al., 2018). Furthermore, there is some evidence that training may overcome effects that the *BDNF* rs6265 polymorphism has on short-term cortical modulation. McHughen et al. (2011) used a marble navigation task to measure skilled motor learning and transcranial magnetic stimulation mapping to assess short-term cortical motor map plasticity, with participants guiding a marble through a sequence of 9 wells (identified on a computer screen) using the right index finger. This required extensive use of the first dorsal interosseous muscle (the same muscle for which the representation was mapped during transcranial magnetic stimulation sessions). Baseline results indicated the presence of *BDNF* polymorphism-related differences in short-term cortical motor map plasticity, where 30 min of motor activity produced a significant increase in the map area for GG homozygotes (Val/Val subjects), but not for carriers with at least one A allele (AG heterozygotes or AA homozygotes). Later, after 12 days of intensive practice, it was observed that the cortical motor map area did not vary in relation to the genotype. In this case, polymorphism effects occurring with short-term cortical plasticity were not found with regard to the long-term cortical plasticity. Moreover, Kleim et al. (2006) showed that corticospinal output of the BDNF in AA homozygotes increased after fine motor training, while subjects with AG or GG genotypes showed opposite effects. This evidence strongly suggests that the effect of the polymorphism occurs as a response of motor training practice. In relation to our findings, this suggests that the practice of sport specific movements may overcome polymorphism-related differences which occurred in the slower, relatively less-trained group of children.

Notably, there are some lines of evidence indicating that the *BDNF* rs6265 polymorphism influences anthropometry and physiological pathways that may be associated with agility performance. In our study, the analysis of RA in the faster group showed that BMI was a significant predictor of RA (Table 4). The relationship between the c.196G>A (p.Val66Met) *BDNF* polymorphism and BMI was confirmed in relation to the population studied. For example, the AA genotype was linked with a higher risk of being overweight in children and adolescents (Martínez-Ezquerro et al., 2018) and a higher risk of anorexia and bulimia nervosa than the c.196G *BDNF* allele carriers in five European populations from France, Germany, Italy, Spain and the UK (Ribases et al., 2004). Moreover, the c.196G>A *BDNF* polymorphism may also influence vascular reactivity and autonomic nervous system functions associated with blood pressure and heart rate regulation, e.g., subjects with the c.196A *BDNF* allele show impaired peripheral vascular reactivity after exercise stimulation (Lemos et al., 2016). It seems that the *BDNF* rs6265 polymorphism may play a mediating role in the relationship between anthropometry, physiological pathways and RA performance. To explain this issue further, further study using mediation methodology is warranted.

To the best of our knowledge, this is the first study analysing the association between a specific genetic variant (*BDNF* rs6265) and sensorimotor adaptation that is based on perceptual and cognitive factors in gross motor skills. In previous studies, sensorimotor adaptability referred mainly to visuomotor tasks, and fine rather than gross motor skills, such as tracking moving objects (Gonzalez-Giraldo et al., 2016; Joundi et al., 2012), finger tapping and pegboard scores (Kleim et al., 2006), or using goggles that left-right mirror-reversed the subject's field of view (Barton et al., 2014). Several studies have shown an association between the c.196G>A (p.Val66Met) *BDNF* polymorphism and impaired perceptual and cognitive function, such as the working memory network, spatial localization, attention and perceptual speed, where the Met allele (A allele) carriers presented higher deficits and reduced hippocampal activity (Schofield et al., 2009). It has been observed that subjects with at least one A allele (subjects with AA or AG genotype) had lower hippocampal volumes than wild-type homozygotes (GG homozygotes). In children, neuro-anatomical differences in the prefrontal cortex, parietal lobes, lateral occipital area and the hippocampus, associated with the *BDNF* polymorphism (homozygous GG carriers vs. AA and AG carriers), were also revealed (Jasińska et al., 2017).

It is important to note certain aspects that may limit interpretation of the present findings. First, we used a single task for the analysis of sensorimotor adaptation, and a cross-sectional design for the study, thus establishment of a clear causal relationship between the long-term effects of team sports training and sensorimotor adaptation is precluded. Second, because of the relatively low number of participants, the influence of the c.196G>A *BDNF* polymorphism on SA should be treated with caution. However, the frequency of the minor c.196A *BDNF* allele (13.5%) in our group of 85 young Polish people of European descent was close to values reported previously in Poles (17.0%) (Wiłkość et al., 2016). Additionally, analysis of genetic and epigenetic variants in other candidate molecules involved in synaptic plasticity, other than the c.196G>A *BDNF* polymorphism mechanism, will be fundamental for a better understanding of SA in reactive environmental tasks in youth athletes.

In conclusion, our findings show that sensorimotor adaptability in reactive agility tasks is mainly dependent on gender and maturity, and can be improved through systematic training. The *BDNF* rs6265 polymorphism may be considered a contributing factor for SA variability in the initial stages of training. The polymorphism-related differences blur following ongoing participation in sports training. From a practical perspective, this type of research can be used to determine if a particular genotype can contribute to an athlete's faster processing speed and better reactive agility training adaptation. This creates the possibility of developing training programs tailored to the individual needs of youth athletes. This study also warrants further investigation using a larger sample size, genotyping of variants other than the *BDNF*, and functional polymorphisms of genes encoding BDNF receptors.
